# Patch Track Software for Measuring Kinematic Phenotypes of Arabidopsis Roots Demonstrated on Auxin Transport Mutants

**DOI:** 10.3390/ijms242216475

**Published:** 2023-11-18

**Authors:** Ashley R. Henry, Nathan D. Miller, Edgar P. Spalding

**Affiliations:** Department of Botany, University of Wisconsin, Madison, WI 53706, USAndmiller@wisc.edu (N.D.M.)

**Keywords:** cell expansion, computer vision, high-throughput phenotyping, image analysis, kinematics, root elongation

## Abstract

Plant roots elongate when cells produced in the apical meristem enter a transient period of rapid expansion. To measure the dynamic process of root cell expansion in the elongation zone, we captured digital images of growing Arabidopsis roots with horizontal microscopes and analyzed them with a custom image analysis program (PatchTrack) designed to track the growth-driven displacement of many closely spaced image patches. Fitting a flexible logistics equation to patch velocities plotted versus position along the root axis produced the length of the elongation zone (mm), peak relative elemental growth rate (% h^−1^), the axial position of the peak (mm from the tip), and average root elongation rate (mm h^−1^). For a wild-type root, the average values of these kinematic traits were 0.52 mm, 23.7% h^−1^, 0.35 mm, and 0.1 mm h^−1^, respectively. We used the platform to determine the kinematic phenotypes of auxin transport mutants. The results support a model in which the PIN2 auxin transporter creates an area of expansion-suppressing, supraoptimal auxin concentration that ends 0.1 mm from the quiescent center (QC), and that ABCB4 and ABCB19 auxin transporters maintain expansion-limiting suboptimal auxin levels beginning approximately 0.5 mm from the QC. This study shows that PatchTrack can quantify dynamic root phenotypes in kinematic terms.

## 1. Introduction

Much of what we know about the control of root growth has come from investigations of Arabidopsis mutants. Studies of the affected genes and the proteins they encode have identified factors that control the size of the root apical meristem, the location of the meristem/elongation zone boundary, and ultimate effects on root length [1]. For example, the size of the root meristem endodermally expressed SCARECROW regulates ARABIDOPSIS RESPONSE REGULATOR1 (ARR1) levels, which has been previously found to regulate the balance between cell division and differentiation [2]. Another study found that the indeterminate growth of a primary root is regulated by PIN2/EIR1 and CONSTITUTIVE TRIPLE RESPONSE1 (CTR1). In *ctr1-1 eir1-1* knockouts, the primary root had no meristematic or elongating tissue, and cells differentiated into xylem tissue and root hairs at the root tip [3]. Similarly, Mandal et al. found that RAV1 regulates the meristem size through the mediation of cytokinin signaling [4]. Studies such as these typically rely on measurements of cell-length profiles and/or reporter gene activity to determine where the elongation zone begins and where cell expansion transitions to differentiation in the maturation zone.

These standard methods do not generate as much insight as methods that quantify the spatial distribution of cell expansion rates in kinematic terms. For example, a mutant’s root may be shorter than the wild type because it has a short elongation zone. Alternatively, the size of the zone could be normal, but the rate of cell expansion within it is low. Kinematics provides an analytical framework to investigate this cellular expansion from the perspective of cells flowing from the root tip. A kinematic analysis can determine if a difference in root growth is due to a difference in elongation zone length, a difference in maximum elongation rate within the zone, or a combination of the two. In the mid-20th century, researchers began to devise ingenious opto-mechanical and photographic methods to measure the displacement of material points along the root [5,6]. The movement of the points, which could be endogenous features such as the end walls of cells or externally applied ink dots, are the data needed for kinematic analysis. The results showed that the local rate of material expansion along the root axis accelerates from almost nothing in the meristem to 40% or more per hour in the elongation zone before rapidly declining to zero in the maturation zone. This is true for large (maize and bean) or small (Arabidopsis) species [7]. Kinematic analyses could be combined with molecular and genetic studies of transcription factors and hormones to create a more detailed description of the mechanism that produces the abrupt acceleration as cells exit the meristem and the similarly abrupt deceleration at the edge of the maturation zone.

Ideally, a kinematic analysis of growth is based on measurements of a large number of infinitesimal pieces of material (particles) moving within a defined frame of reference. In the case of a root, the quiescent center (QC) in the meristem is a convenient origin for the reference frame. From the perspective of an observer at the root tip, a distant particle would appear to move away due to the expansion of the material between the QC and the particle. A particle closer to the observer would move away more slowly due to the lesser amount of expanding material in the interval between this closer point and the observer. The movements of these particles relative to the observer during a time interval form a Lagrangian description of growth.

Erickson and Sax [5], and Gandar [8,9] established the mathematical framework for describing the motion of growing root cells in the Lagrangian reference frame. Morris and Silk [10] showed that a flexible logistic function effectively fits the particle velocities when plotted versus the distance from the root apex. The first derivative of the fitted function produces the bell-shaped relative elemental growth rate (REGR) profile that describes the size, shape, and position of the elongation zone along the length of the root. The advent of digital imaging devices spurred the development of software for producing kinematic analyses. For example, KineRoot analyzed externally applied specks of carborundum powder to track points needed for a kinematic analysis of bean root growth [11]. Baskin and Zelinsky developed Stripflow, which tracked patches of endogenous texture to determine the velocity profile along the root [12]. Zheng et al. used a texture-tracking approach to show that *AUXIN UP-REGULATED F-BOX PROTEIN1* (*AUF1*) mutant roots displayed shorter roots than wild-types after cytokinin treatment due to lower growth rates, a lower maximum REGR, and a shorter elongation zone [13]. 

The methods for tracking material particles as they flow from the root tip are based on the concepts of optic flow as implemented by Lucas and Kanade [14]. Fleet & Weiss [15] expanded this work by including spatial intensity gradients of nearby pixels to form a patch of material to track. To follow the correct patch through the image frames, they placed a constraint requiring the spatial intensity gradients of the first patch to be equal to the spatial intensity gradients of the same patch in the subsequent frame. Using a least-squares estimator, a match is found when the constraint errors are minimized [14,15]. Once a match is found, the velocity of the patch between frames can be calculated.

The present study used the concepts and techniques of optical flow and kinematics to produce a new tool for measuring root growth. We chose to demonstrate its use on auxin transport mutants because auxin is known to affect root growth and development. For example, during gravitropism, auxin gradients establish the difference in the rate of cellular expansion on either side of the root that produces curvature [16]. Also, auxin inhibits root cell expansion by inducing alkalization of the apoplast [17]. Other studies have shown that auxin alters functional boundaries due to its role in promoting cell division and inhibiting expansion [18,19]. Some studies indicate that the meristem/expansion transition correlates with the tip-high auxin gradient along the root [20,21,22].

Auxin transport proteins help to form auxin gradients by mediating separate shootward and rootward flow paths. One member of the highly studied PIN-FORMED (PIN) family of membrane-bound auxin efflux transporters, PIN2, is responsible for shootward auxin flow and is polarly expressed in the lateral root cap and epidermis in the vicinity of the meristem-elongation zone boundary [22,23]. Mutations in *PIN2* result in shorter roots and reduced meristematic zones [21]. Membrane proteins in the B subfamily of ATP-binding cassette transporters (ABCB) also mediate auxin transport in the root [24,25,26]. One of these proteins, ABCB4, has a similar expression pattern as PIN2, but the localization extends further shootward into the elongation and maturation zones [27,28]. ABCB4 is responsible for shootward auxin transport. Without ABCB4, shootward auxin flow is only 50% of the wild-type level, while rootward auxin flow is not affected [29]. ABCB19 is a transporter responsible for 80% of rootward auxin flow through the stele, and it appears to also recycle auxin from the cortex back into the stele [29,30]. ABCB19 has no detectable effect on shootward auxin flow [29]. The present work uses a new software tool for measuring root growth in kinematic terms to determine how PIN2, ABCB4, and ABCB19 auxin transporters shape the elongation zone of the Arabidopsis root.

## 2. Results

Kinematics is the study of material motion without concern for the forces that cause it. Its concepts and methods are applicable to the study of root growth [5,6,31]. Optical flow is the study of apparent motion in a time series of images [32]. Here we describe a custom platform for acquiring time series of images and a software tool that uses optical flow concepts [14,15] to produce a kinematic description of root growth.

**Figure 1:**
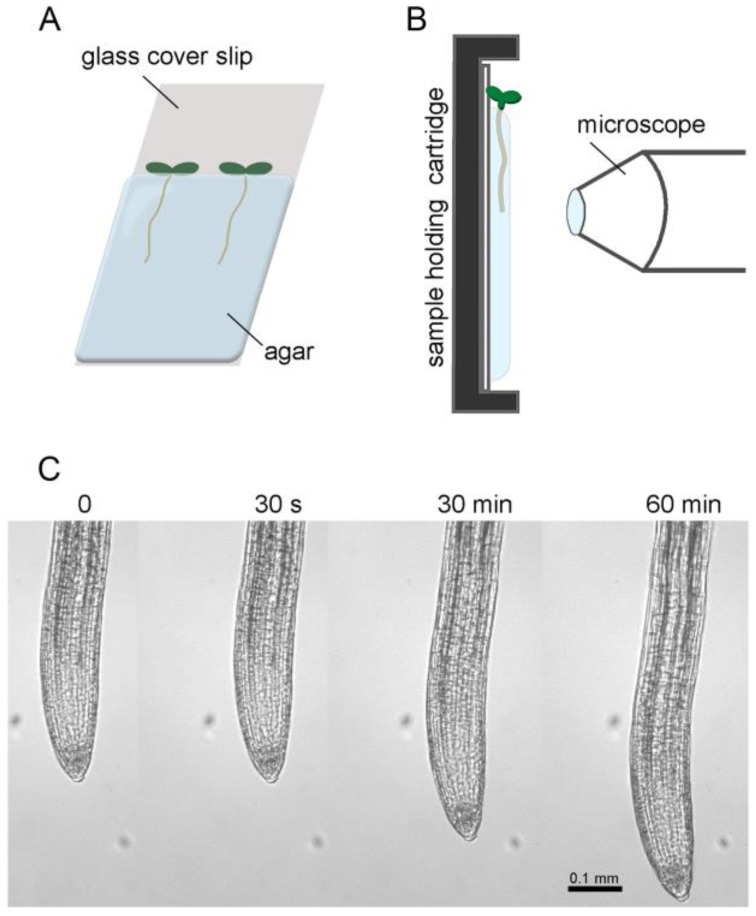
Diagram of the growing and imaging process of the Arabidopsis seedling roots for kinematic analysis. (**A**) Coverslip with seedlings sown into an agar-based medium. (**B**) The coverslip mounted in a 3D-printed cartridge and placed on the stage of a horizontal microscope. (**C**) Four frames from a one-hour time series, where images are taken every 30 s at a resolution of 1500 px mm^−1^.

Figure 1A shows how *Arabidopsis thaliana* seedlings were cultured such that the root grew within a transparent gelled medium and along the surface of a vertical glass coverslip. The coverslip with seedlings was secured in a plastic cartridge that was mounted on the stage of a horizontal compound microscope fitted with a computer-controlled camera rather than an eyepiece (Figure 1B). Images were collected every 30 s for one hour (a total of 120 frames per trial). Figure 1C shows example frames acquired at 0 s, 30 s, 30 min, and 60 min time points.

**Figure 2:**
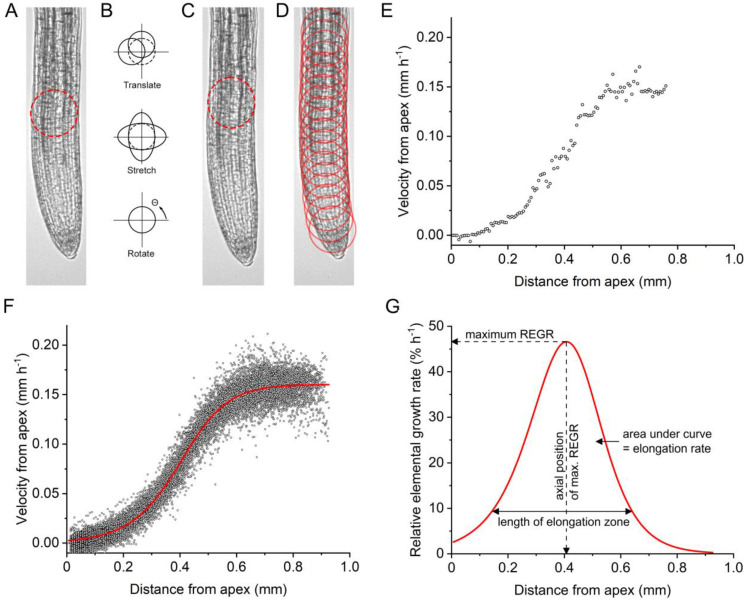
The process of our kinematic analysis pipeline, from root images to extracted features of each root’s growth zone. (**A**–**D**) Image patch tracking as a root elongates. (**A**) Root at 0 min with a disk capturing an image patch at the beginning of the elongation zone. (**B**) Possible disk transformations to match the image patch in A to the sequential image patch in (**C**). (**C**) Root at 5 min with a translated, stretched, and rotated disk capturing the same image patch as the disk in (**A**). (**D**) Several disks along the root midline, each capturing and tracking their respective image patches as the root elongates. (**E**) Velocity points along the root midline from all disks tracking image patches from one frame to the next. (**F**) Velocity points along the root midline for all frames and all disks. The red line is the fitted velocity profile for that root. (**G**) The red line is the derivative of the velocity curve in F and is the relative elemental growth rate (REGR) curve. The arrows and text denote the four kinematic traits that are extracted from the REGR curve of each root.

To begin an analysis, the user provides the program with a time series of images (image stack) and selects values for the size and number of circular regions (disks) that will be tracked through space and time to produce a kinematic description of the root’s growth. The default values are appropriate for Arabidopsis roots imaged on the platform assembled for this study. A user with different root images should experimentally determine the best disk size and spacing. Next, the user opens the first image in the stack to select 10–12 points along the middle of the root, beginning with the quiescent center at the apical edge of the meristem and ending where the root meets the edge of the frame. The program interpolates 100 evenly spaced points to create a midline. The software automatically places the disks to be tracked at 10-px intervals with their centers on this midline. The user may select a different spacing. The first disk is placed where the user marked the quiescent center (QC). Figure 2D shows an example with only 20 such disks, so they can be seen well.

Each circular image patch defined by a disk in frame *n* will be present in frame *n* + 1. The goal of the algorithm implemented here is to determine how far each patch moved between frames, which requires finding the new location of each disk in the subsequent frame. The growth process may distort a disk while displacing (or translating) it along the root axis, which adds to the technical challenge of matching patches.

The data are pixel intensities (*I*) recorded over time (*t*). *I* varies spatially ($\partial I_{x}$ and $\partial I_{y}$) because endogenous structures in the roots, such as cell walls and intercellular air spaces, are variously opaque to the infrared backlight. Growth imparts velocity to these material marks. The material derivative $D_{t}I$ describes how the intensity changes with respect to time.

(1) $$D_{t}I=\left(\partial I_{x}\cdot v_{x}\right)+(\partial I_{y}\cdot v_{y})+\partial I_{t}=v\cdot \nabla I+\partial_{t}I$$

If we assume that the camera is stationary and that the illumination is constant, then a pixel in frame *n* should have the same intensity in frame *n* + 1 unless growth moves the root material relative to the sensor. If we can account for the movement of material, then the difference in intensity between the corresponding points in frames *n* and *n* + 1 should be zero. We seek the velocity (*v*) for each patch that makes $D_{t}I=0$ between two successive frames. 

As a patch translates along the root midline, it may also stretch due to the expansion of the material within it and rotate if the root is bending (Figure 2B). These deformations must be considered if matching patches based on *D_t_I* = 0 are to be successful. The velocity gradient (∇v) encompasses stretches and rotation around a point *x* that is translating with a velocity *v* (Equation (2)).

(2) $$v\left(x+dx\right)=v\left(x\right)+\nabla v\left(x\right)\cdot dx$$

Consolidating unknowns *v*$\left(x\right)$ and $\nabla v\left(x\right)$ into a transformation, T allows Equation (2) to be written as Equation (3).

(3) $$v\left(x+dx\right)=\left[v\left(x\right),\nabla v\left(x\right)\right]\cdot \left[1,dx\right]=T\left(x\right)\cdot \tilde{dx}$$

Thus, Equation (1) defines the problem to be solved and $D_{t}I=0$ constrains the velocity solution to motion following the material. Equation (2) states a first-order model for velocity, and Equation (3) condenses it. Combining Equations (1) and (3) produces Equation (4), a model for patch motion between two successive frames that can be used to find the values in *T* that make $D_{t}I$ = 0.

(4) $$D_{t}I=\nabla I\cdot T\cdot \tilde{dx}+\partial_{t}I=\left(\nabla I\;⊗\;\tilde{dx}\right)\cdot T+\partial_{t}I$$

Equation (4) indicates that the tensor product (⊗) of the gradient of image intensity at each point in the image patch (∇I) and $\tilde{dx}$ (pixel coordinates within the patch relative to the center point), dotted with a vectorized *T*, plus the difference in pixel intensity between the two time points ($\partial_{t}I$) equals $D_{t}I$, which is constrained to zero. All terms but *T* are measured from the images. Solving for *T* will determine how the patch in image n must morph and shift to match a patch in image *n* + 1. To solve for *I*, we initialize it to the identity transformation (I), evaluate the resulting change in intensity for each pixel surrounding *x*, and solve for *dT* (Table 1). The sum of *dT* and *T^n^* is *T^n^*^+1^. The program performs steps 2–4 with *T^n^*^+1^, and this process repeats until the Euclidian norm of *dT*, which encompasses rotation, stretch, and translation, is less than a threshold of 10^−6^ pixels. At this point, a match has been found between two image patches within disks on subsequent frames. In a typical case, six or fewer iterations are required to reach this threshold.

The above text describes the process of matching one disk with the same disk on a subsequent image frame (Figure 2A–C). The program then calculates the velocity of the disk center point based on the difference between its location in frames *n* and *n* + 1. Following the best matching of patches from one frame to the next, the velocity of the disks’ center points with respect to the quiescent center can be calculated. Every disk’s center-point velocity calculated between frames 0 and 1 for the example root (Figure 2D) is plotted as a function of axial position in Figure 2E. 

The program repeats this process with frame *n* + 1. New circular disks are placed on the image and will follow that patch to frame *n* + 2, and so on until the patches are followed through all 120 frames. Velocities are calculated with each iteration through consecutive frames to produce approximately 15,000 separate velocity measurements at points all along the midline (Figure 2F). For each completed analysis, the program stores the value pairs (velocity and position) in units of pixels/frame and pixels from the quiescent center, respectively, in a comma separate values (csv) file. A user converts the values to millimeters (mm) and hours (h) using an empirically determined resolution factor specific to the platform and the frame acquisition rate. An appropriate frame rate will be slow enough that material expands enough during the interval to be measured, but fast enough that disks do not enter a substantially different region of the growth zone during the interval. For Arabidopsis roots, 30 s is an appropriate time step at the resolution achieved with the platform used here, which was 1450 px mm^−1^. The program also returns a plot similar to that shown in Figure 2F for a simple assessment of quality. The program also returns csv files of the fitted velocity and relative elemental growth rate (REGR) data, the maximum REGR value, the axial location of the maximum REGR, the length of the growth zone, and the coordinates of the disks for each frame.

The sigmoidal red line in Figure 2F is the best fit of the flexible logistic function (Equation (5)) that Morris and Silk [10] derived and effectively used to analyze similar velocity profile data obtained by manually measuring photographs of hand-marked maize roots [33].

(5) vx=vf[1+e−kx−x0]1n

We used the Nelder-Mead method () in the R software package (https://CRAN.R-project.org/package=neldermead) [34,35] to find the values for *v_f_, k*, *x*_0_, and *n* that gave the best fit for each point cloud. Differentiating the velocity profile curve gives the relative elemental growth rate (REGR) profile, shown in Figure 2G. In this example, the peak REGR is approximately 45% h^−1^, very similar to other measurements of Arabidopsis roots at 40–50% h^−1^ [7,36] and, notably, the much larger maize root [5,37] and bean root [11]. Relative elemental growth rate, a measurement of local material expansion rate, is not a function of organ size.

### 2.1. Root Growth Zone Traits

The four traits that describe root growth in kinematic terms can be derived from the REGR profile curve and are labeled in Figure 2G. Maximum REGR is the highest rate observed, the peak of the REGR profile. The axial position where the root experiences the highest relative growth rate is a second trait that helps to characterize the root. The average growth rate during the hour-long observation is the area under the curve and is also *v_f_*. Lastly, we define the elongation zone as the length of the root in which REGR exceeds 20% of its maximum value. Each of these four kinematic traits can be calculated directly from the best fit of Equation (5) to the velocity profile results the program produces. 

### 2.2. Auxin Transport Plays a Role in Defining the Growth Zone

For this tool to be useful, it must be able to quantify differences caused by mutations or treatments that researchers use to investigate the mechanisms of root growth control. Auxin is a plant hormone known to control root growth. It is unique among root growth regulators in having a transport mechanism that directs its flow through tissues. Auxin moves toward the root tip through the middle of the root (stele and possibly cortex) and back toward the shoot through the outer cell layer (lateral root cap and epidermis). Mutations in the PIN and ABCB transporter genes selectively disrupt these rootward and shootward transport pathways. We used *abcb4*, *abcb19*, and *pin2* mutants to determine if the image acquisition and analysis platform we developed could resolve kinematic phenotypes due to altered auxin transport through roots.

### 2.3. REGR Profiles of Auxin Transport Mutants

**Figure 3:**
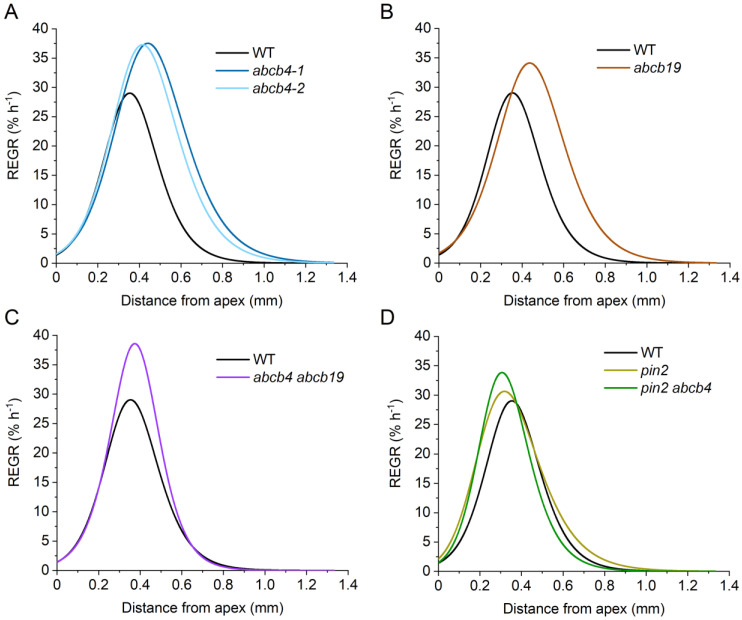
REGR profiles of the auxin transport mutants. (**A**–**D**) REGR profiles of *abcb4*, *abcb19*, *abcb4 abcb19*, *pin2,* and *pin2 abcb4* mutants with their Col-0 wild types, respectively. (**A**) Two alleles of the *abcb4* mutation. In each experiment, 13 to 18 primary roots were analyzed.

**Figure 4:**
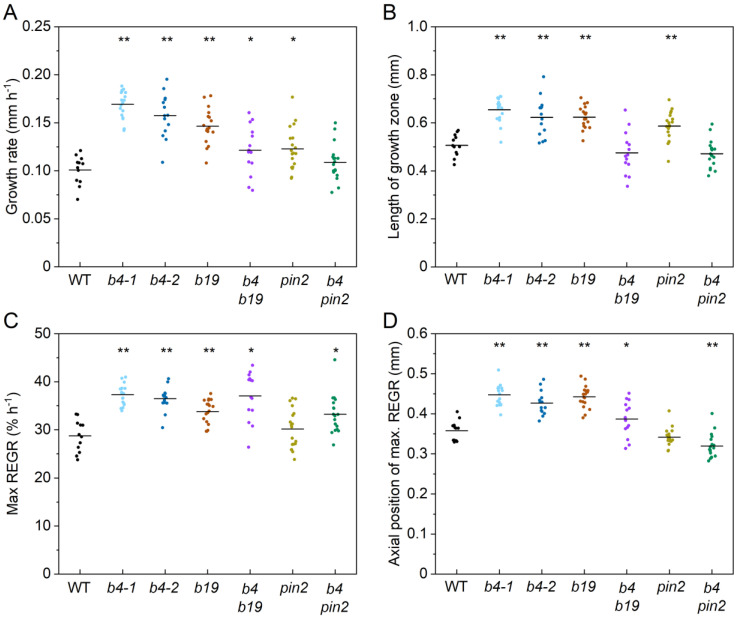
Distribution of growth zone traits by genotype. (**A**–**D**) Final growth rate, length of growth zone, max REGR, and position of max. REGR differences between the WT and auxin transport mutants, respectively. In each experiment, 13 to 18 primary roots were analyzed. ** denotes *p*-value < 0.001 and * denotes *p*-value < 0.01.

Two independent *abcb4* alleles, previously shown to have substantially reduced shootward flow of auxin in roots [29], similarly increased the length of the elongation zone, increased overall growth rate, increased maximum REGR, and shifted the location of maximum REGR shootward compared to the wild type. Figure 3A shows the REGR profile curves, and Figure 4 shows the values of the extracted kinematic traits. However, these *abcb4* mutations had little effect on the location or shape of the apical edge of the growth zone. The *abcb4* mutants differ from the wild type most substantially on the basal side of the elongation zone. This indicates that ABCB4 shapes the basal side of the elongation zone.

The ABCB19 transporter plays a major role in moving auxin rootward, through the central tissues to the tip, and it may ‘recirculate’ auxin from the epidermis into the central cylinder in the elongation zone [29]. An *abcb19* mutant, like *abcb4*, displayed a significantly higher growth rate, a higher maximum REGR, a longer elongation zone, and a more basal (shootward) position of the maximum REGR. As in *abcb4*, *abcb19* roots grew faster than the wild type due to material remaining in an elevated expansion phase at greater distances from the apex. The results did not match our expectation that *abcb4* and *abcb19* mutants would display opposite phenotypes due to their established roles in oppositely directed auxin transport streams. If *abcb4* and *abcb19* mutations affect growth zone dynamics by independent means, then a double mutant may be expected to display a more severe version of their separate and similar phenotypes. However, the *abcb4 abcb19* mutant we constructed displayed a more wild-type growth zone. Each of the single mutant kinematic phenotypes was reduced or not significant in the case of the double mutant.

PIN2 is another auxin transporter that, like ABCB4, moves auxin shootward through the lateral root cap and epidermal cell layers [38]. We determined that a *pin2* mutant had a statistically higher growth rate and a longer elongation zone than the wild type (Figure 4). However, the maximum REGR and position of the maximum REGR were not different from the wild type. The faster growth rate of *pin2* roots is apparently due to a longer elongation zone and not because the material has a greater peak capacity to expand. When we knocked out both *PIN2* and *ABCB4*, we observed a higher maximum REGR and a shift in the location of this peak REGR closer to the root tip than the wild type. The other two kinematic traits were not different from those of the wild type. A summary of the kinematic effects of the auxin transport mutations and some possible explanations are presented in the Discussion Section.

## 3. Discussion

Of the previously published image analysis programs designed to measure root growth as a kinematic or flow process, our PatchTrack has most in common with Stripflow [12], which is a successor to the successful RootFlowRT software [36]. Stripflow, like PatchTrack, finds a small region of root in a succeeding image frame, determines the displacement (translation) it underwent, and then calculates the axial component of that small element’s velocity. In Stripflow, the regions to be matched in the second image are defined by narrow rectangles (strips), each oriented perpendicular to the midline and centered at a different point on the midline. The PatchTrack algorithm operates on disks, which it translates, stretches, and rotates to find the best matching image patch in the subsequent frame. Incorporating rotation uniquely enables PatchTrack to measure the axial velocity of an element even if the roots were bending during the observation period. Stripflow was designed to process two successive images, which Iwamoto et al. [39] refer to as the source image and the destination image in the description of their GrowthTracer program for kinematic analysis of root growth. (GrowthTracer uses a largely unrelated method for identifying matching regions in the source and destination images.) Unlike Stripflow or GrowthTracer, PatchTrack was designed to analyze not a single pair of source and destination images but each sequential pair in a series of 120 frames collected over 1 h of growth. Repeated sampling of at least 80 disks per root creates a dataset large enough to support the accurate fitting of a suitable model (the flexible logistics equation) to the consistently sigmoidal velocity profile (Figure 1F). The velocity profiles are detailed enough to distinguish the effects of mutations on kinematic traits (Figure 4).

PatchTrack requires a user to register by clicking on the locations of the QC. Variation in the choice of the QC anchor point would not significantly affect the results of spatial traits. Natural variation between roots in a trait such as the position of maximum REGR (Figure 4C) is orders of magnitude greater than a few or a great many pixels differences in QC selection because each pixel represents a distance of only 0.7 μm. The user also seeds the algorithm by selecting approximately 10 points near or on the midline in the first image of the series. These manual steps do not significantly slow the analysis of a time series (stack), which subsequently runs automatically to completion in 3–5 min. No splicing of images or preprocessing steps are needed. The seedling is not transplanted or otherwise manipulated because the seeds are sown and cultured directly in the sample holder. Because endogenous root features create the variation in pixel brightness that the algorithm uses for tracking, there is no need to apply fiducial marks such as carborundum powder [11]. Efficiency and throughput make PatchTrack suitable for analyzing large numbers of trials required for genetic studies of the root growth engine.

In most respects, Stripflow, GrowthTracer, and PatchTrack generate similar descriptions of the Arabidopsis root growth engine. Each shows that cells elongate slowly until they become approximately 100 μm removed from the quiescent center. From this point, REGR dramatically rises, reaching 30–50% h^−1^ at a position 350–600 μm from the quiescent center, depending on the study. Where on the root axis a material element begins to increase its rate of expansion (accelerate) is approximately where the apical meristem ends and the elongation zone begins. Where a material element’s rate of expansion approaches zero, it is approximately at the end of the elongation zone and the beginning of the maturation zone. The sigmoidal shape of the velocity profile that PatchTrack generates indicates that these transitions are smooth, continuous processes. Stripflow generates velocity profiles that have been approximated by a slow linear phase in the meristem and a fast linear phase in the elongation zone. The discontinuity between the two slopes has been used to demarcate the boundary between the meristem and the elongation zone [40,41]. The most common method of determining the boundary between the meristem and the elongation zone relies on manual inspection of cell lengths to identify the spot where cell elongation first becomes evident. Salvi et al. [1] and Pacifici et al. [42] discuss the difficulties and shortcomings of this and other methods for identifying the boundary. The velocity data that PatchTrack generates are consistent with continuous, though abrupt, changes in the rate of local expansion at the boundaries of the elongation zone. A discrete boundary is not discernible within a region of continuously changing axial velocity. Therefore, we chose the points at which REGR equals 20% of its peak value as the apical and basal extents of the elongation zone. While arbitrary, this value allowed a systematic and objective comparison of mutants to the wild type.

When grown in the sample fixture mounted on the stage of the microscope and illuminated with white light (i.e., possibly non-ideal conditions), a cell that is 150 μm from the quiescent center (QC) in a wild-type root (Col-0 ecotype) expands 8.6% h^−1^ (Figure 4A). When that same cell occupies a place 300 μm behind the QC, it expands 26% h^−1^. Thus, only two or three cells in a file may separate regions of a 3-fold different REGR. There may be no other example in the plant in which a cell undergoes as large an acceleration and deceleration as at the apical and basal ends, respectively, of the elongation zone.

Most of the ideas about the mechanism responsible for the boundary between the meristem and the elongation zone include a role for auxin. Elevated auxin in the root apex affects meristem size by promoting cell division and inhibiting cell expansion [18,19]. The relatively high concentration of auxin in the root apex probably results from a combination of the lateral root cap acting as an auxin sink, PIN and ABCB-mediated transport, and local biosynthesis and degradation of auxin [20,21,22,29,43,44]. A decline in auxin concentration with distance from the tip is a component of a molecular network in which PLETHORA transcription factors negatively regulate ARABIDOPSIS RESPONSE REGULATORS to establish the meristem/elongation zone boundary [45,46,47]. In addition to establishing the position of the boundary, auxin plays a role in determining the rate of cell expansion within the elongation zone. Auxin increases the rate of cell expansion in accordance with the acid growth hypothesis [17] but becomes inhibitory at supraoptimal concentrations [48,49]. For PatchTrack to be useful in studies of the Arabidopsis root growth engine, it should be able to detect the effects of altered auxin transport in the root apex. The results in Figure 3 and Figure 4 demonstrate this is true.

ABCB4 promotes shootward auxin transport through, and out of, the elongation zone, primarily in the epidermis. Conversely, ABC19 promotes rootward auxin flow primarily through central cell files and appears to promote the recirculation of some of the shootward stream back into the central rootward stream [29,30]. Despite ABCB4 and ABC19 participating primarily in opposite auxin transport streams, *abcb4* and *abcb19* mutants displayed similar phenotypes: the growth zones extended further shootward than wild type (Figure 3A,B and Figure 4B,D) and maxREGR was higher (Figure 3A,B and Figure 4C) in both mutants. To explain these results, we propose that ABCB4 normally maintains auxin at suboptimal levels in the region between 0.4 and 1 mm from the QC by promoting its shootward movement. The higher REGR between 0.4 and 1 mm in an *abcb4* mutant would therefore result from elevated auxin levels in this region. In *abcb19*, we propose that higher auxin levels in the outer cell layers, which Lewis et al. [29] suggested were a result of impaired recirculation, promote cell expansion in the region between 0.4 and 1 mm. The *abcb4 abcb19* phenotype did not resemble either the single mutant phenotype or their sum (Figure 3C) but instead displayed a more wild-type growth zone length and position (Figure 4B,D). We propose that the combination of impaired shootward flow and impaired retrieval/recirculation elevates auxin to supraoptimal levels in the region between 0.4 and 1 mm from the QC, thereby suppressing REGR to create a profile similar to the wild type. Thus, the decline in the rate of cell expansion on the shootward side of the REGR profile may reflect supraoptimal auxin in the double mutant but suboptimal auxin concentration in the wild type. Detailed measurements of auxin patterns in these mutants could test this hypothesis.

Unlike the *abcb* mutants, a *pin2* mutation lengthened the expansion zone (Figure 3D and Figure 4B), apparently by shifting one boundary rootward and the other shootward. If PIN2 moves auxin from the meristem to create a supraoptimal auxin concentration at the apical boundary of the elongation zone, the boundary should shift rootward in a *pin2* mutant, as observed. Published protein and mRNA expression patterns indicate that PIN2 acts closer to the tip than ABCB4 [21,50]. Using the gene expression data from Li et al. [51], we determined that PIN2 mRNA is 1.5-fold higher in the meristem than in the elongation zone of Arabidopsis roots, while ABCB4 mRNA is 5.2-fold higher in the elongation zone than in the meristem. If PIN2 is important for moving auxin out of the meristem and ABCB4 plays a larger role in moving auxin out of the elongation zone, then impairment of the former would reduce the effect of additionally impairing the latter. The *abcb4 pin2* and *pin2* phenotypes are very similar, consistent with this prediction (Figure 3D and Figure 4D). Thus, it appears that PIN2-mediated auxin flow affects the apical boundary of the root elongation zone and ABCB4 affects the basal boundary. This kinematic analysis discovered new phenotypes of well-studied auxin transport mutants, demonstrating the usefulness of the PatchTrack pipeline in studies of the root growth engine.

## 4. Materials and Methods

### 4.1. Plant Material and Growth Conditions

Two milliliters of melted 1% agarose media containing 1 mM KCl, 1 mM CaCl_2_, 5 mM 4-morpholineethanesulfonic acid (pH corrected to 5.7 with Bis-Tris Propane) was spread over a 22 mm × 40 mm coverslip and allowed to solidify. One-third of the media was cut to produce a shelf on which 3–4 Arabidopsis seeds were sown (Figure 1A). Coverslips with seeds were placed in square Petri plates containing the same medium to maintain humidity and stored in the dark at 4 °C for 2–4 days. After the stratification treatment, the plates were transferred to a growth chamber and kept under constant light, 21 °C, 63% relative humidity, and at an 80° angle to promote root growth along the coverslip surface. The seedlings grew for 3 days before being placed in the imaging pipeline.

The plant material used was Col-0 for the wild type, *pin2*, *abcb4-1*, *abcb4-2*, and *abcb19-3*. The double mutants used in this study (*abcb4 abcb19* and *abcb4 pin2*) were newly created by crossing and confirmed by PCR as described in Lewis et al. [29]. PCR for *pin2* plants used the following genotyping primers, T-DNA primer LB1a (5′-TGGTTCACGTAGTGGGCCATCG-3′) and the gene-specific PIN2 F (5′-TGATGTTGTTGATCATTTTATGGGACC-3’) and PIN2 R (5′-CCTTAGGGCCATCGCAAACCC-3’).

### 4.2. Image Collection

A coverslip with seedlings was attached to a custom-printed, open-frame cartridge that fit onto the stage of a horizontal compound microscope. The microscope was fitted with a complementary metal-oxide semiconductor (CMOS) camera (Flea3 GigE-50S5M, Point Grey Research North Billerica, MA, USA) in place of the eyepiece and a 10× objective lens. A computer running Point Grey FlyCapture2 Camera Selection software (v. 2.5.2.3) controlled the camera to capture images of a single growing root every 30 s for 1 h, producing a time series of images. Each image was saved as a tagged image format (TIF) file and has a resolution of 1450 pixels mm^−1^. The resolution gave ample texture to measure patches along the root, and running five samples at once produced a high-throughput pipeline. Five identical microscope imaging platforms operated in parallel to achieve the data collection throughput this study required.

## Figures and Tables

**Table 1:** Pseudo-code for solving for *T*.

Steps	Mathematical Function
step 0: initialize 1	$T^{0}=I$
step 1: evaluate	$M=\left(\nabla I⊗dx\right)$
while *dT* > threshold	
step 2: evaluate intensity change	$D_{t}I=I\left(T^{n}\cdot dx+x,t+1\right)-I\left(x,t\right)$
step 3: solve for change in *T*	$dT=-D_{t}I*{\left(M\right)}^{†}$
step 4: update *T*	$T^{n+1}=T^{n}+dT$

## Data Availability

The computer code for PatchTrack is available at https://github.com/phytoMorph/phytoMorph_kinematics.
